# Convergent and divergent evolution of genomic imprinting in the marsupial *Monodelphis domestica*

**DOI:** 10.1186/1471-2164-13-394

**Published:** 2012-08-16

**Authors:** Radhika Das, Nathan Anderson, MaryEllen I Koran, Jennifer R Weidman, Tarjei S Mikkelsen, Michael Kamal, Susan K Murphy, Kerstin Linblad-Toh, John M Greally, Randy L Jirtle

**Affiliations:** 1 Department of Radiation Oncology, Duke University Medical Center, Box 3433, Durham, NC, 27710, USA; 2 Broad Institute of MIT and Harvard, Cambridge, MA, 02142, USA; 3 Department of Obstetrics and Gynecology, Duke University Medical Center, Durham, NC, 27708, USA; 4 Department of Medical Biochemistry and Microbiology, Uppsala University, Uppsala, 75123, Sweden; 5 Department of Medicine (Hematology) and Molecular Genetics, Albert Einstein College of Medicine, Bronx, NY, 10461, USA; 6 McArdle Laboratory for Cancer Research, Department of Oncology University of Wisconsin-Madison, 1400 University Avenue, Madison,, WI 53706, USA

**Keywords:** Genomic imprinting, Marsupials, Eutherians

## Abstract

**Background:**

Genomic imprinting is an epigenetic phenomenon resulting in parent-of-origin specific monoallelic gene expression. It is postulated to have evolved in placental mammals to modulate intrauterine resource allocation to the offspring. In this study, we determined the imprint status of metatherian orthologues of eutherian imprinted genes.

**Results:**

*L3MBTL* and *HTR2A* were shown to be imprinted in *Monodelphis domestica* (the gray short-tailed opossum). *MEST* expressed a monoallelic and a biallelic transcript, as in eutherians. In contrast, *IMPACT, COPG2,* and *PLAGL1* were not imprinted in the opossum. Differentially methylated regions (DMRs) involved in regulating imprinting in eutherians were not found at any of the new imprinted loci in the opossum. Interestingly, a novel DMR was identified in intron 11 of the imprinted *IGF2R* gene, but this was not conserved in eutherians. The promoter regions of the imprinted genes in the opossum were enriched for the activating histone modification H3 Lysine 4 dimethylation.

**Conclusions:**

The phenomenon of genomic imprinting is conserved in Therians, but the marked difference in the number and location of imprinted genes and DMRs between metatherians and eutherians indicates that imprinting is not fully conserved between the two Therian infra-classes. The identification of a novel DMR at a non-conserved location as well as the first demonstration of histone modifications at imprinted loci in the opossum suggest that genomic imprinting may have evolved in a common ancestor of these two Therian infra-classes with subsequent divergence of regulatory mechanisms in the two lineages.

## Background

Genomic imprinting is an epigenetic phenomenon characterized by the monoallelic expression of a gene in a parent-of-origin dependent manner [1]. Its discovery in mammals showed that parental genomes are functionally non-equivalent, and that the sex of the parent, not of the resulting progeny, is critical in determining expression of a particular allele [2-4].

In vertebrates, imprinting is believed to have originated approximately 150 million years ago, in the common ancestor of the two Therian infra-classes: eutherians (placental mammals like humans and mice), and metatherians (marsupials like the opossum and tammar wallaby which have a less invasive placenta) [5]. The evidence comes from approximately 98 imprinted genes that have been discovered to date in eutherian mammals, and the six known to be imprinted in the metatherians [6-12]. Imprinted genes have not been identified in prototherian (i.e. platypus and echidna) and avian (i.e. chicken) species [6,13]. To explain this unique phylogenetic distribution of imprinted genes, the “Conflict Hypothesis” proposes that genomic imprinting evolved in placental mammals in response to polygamy, viviparity, and multiple births [14,15]. This theory is based on maximizing competitive fitness of the father’s progeny while preserving the ability of the mother to equally provide care to all her offspring, regardless of paternity. A corollary to this hypothesis is that paternally and maternally expressed genes will enhance and inhibit growth, respectively.

An unusual feature of imprinted genes in eutherians is that they tend to occur in clusters throughout the genome. This suggests that shared regulatory elements play a role in epigenetic control of these clusters. In many imprinted gene clusters, imprint control regions (ICRs) function as discrete *cis*-acting DNA elements that are characterized by heritable epigenetic marks that distinguish the two parental alleles [16]. These ICRs simultaneously and often reciprocally regulate two or more imprinted genes.

The imprint mark in eutherians includes germ-line derived cytosine methylation patterns wherein the two alleles exhibit opposite methylation states [17]. A group of such differentially methylated CpG sites at a locus constitute a differentially methylated region (DMR). The methylation marks are completely erased in the germ cells and then re-established in the gametes, based on the sex of the individual now carrying the DNA. Surprisingly, of the six genes known to be imprinted in metatherians, DMRs are only present at *PEG10*[7] and *IGF2*[18]. Moreover, the presence of a DMR is not sufficient to confer imprinted expression in eutherians, since *IGF2R* is biallelically expressed [6] in humans despite having a DMR in intron 2 [19].

Differential chromatin states of the parental alleles resulting from covalent modification of histone tails also plays a prominent role in regulating expression and imprinting patterns. Repressive modifications, including H3 Lysine 9 (H3K9) and H3 Lysine 27 (H3K27) methylation, function in silencing of an allele whereas active marks, including H3 Lysine 4 (H3K4) methylation and histone acetylation are associated with the expressed allele. These histone modifications at the promoter region of imprinted genes are better correlated with expression than the methylation marks [20-22]. As a case in point, human *IGF2R* has an intronic DMR in peripheral tissues, but lacks the promoter-restricted histone code present in the mouse, and is biallelically expressed. Thus the histone code may be the “primordial imprint mark,” and mechanisms of DNA methylation were probably added later to assist in stabilizing the expression status [23].

Understanding the molecular mechanisms by which imprinting is established in all mammals is critical to furthering knowledge about imprint gene regulation in humans. Although differential methylation of the parental alleles is involved in controlling genomic imprinting in eutherians, there is no clear evidence supporting a role for DMRs in metatherian imprinting. The DNA methyltransferases necessary for conferring imprinting are present and functional in the metatherians [24,25], yet DMRs are present at only two of the six currently known imprinted genes. The lack of DMR identification at the other imprinted loci may reflect an inability to thoroughly investigate the sequence content associated with these regions in metatherians due to incomplete sequence availability at the time these studies were done. Alternatively, it may be due to the true absence of DMRs in these regions if imprinting is indeed controlled primarily by histone marks in metatherians. The sequencing of the genome of the gray short-tailed opossum *Monodelphis domestica(M. domestica)* now allows for the comprehensive investigation of such imprint regulatory regions.

It is presently unknown if chromatin modifications exist at imprinted loci in metatherians despite the knowledge that histone modifications, and not DNA methylation, are involved in paternal X chromosome silencing in metatherians [26,27]. Since imprinted X-inactivation appears to have co-evolved with genomic imprinting in Therians, it is possible that the same molecular mechanism is used to delineate the active and inactive regions for both of these epigenetic phenomena [23,28].

Here we exploited the availability of the genomic sequence of the gray short-tailed opossum, *M. domestica*, to identify orthologues of known eutherian imprinted genes and then determined their imprint status in this metatherian. CpG rich regions in close proximity to these genes were also identified and examined for the presence of differential methylation. We then assessed active H3K4 dimethylation and repressive H3K9 trimethylation in these regions. We identified a novel DMR within *IGF2R*, but DMRs were not present near or within the other genes imprinted in the opossum. The active alleles of the imprinted genes were enriched for the active mark H3K4 dimethylation. The discovery of the novel *IGF2R* DMR demonstrates that differential methylation might be present at imprinted loci in metatherians at non-conserved locations, but these have been missed in previous analyses due to incomplete sequence availability. Moreover, the presence of histone modifications at imprinted loci also shows that genomic imprinting arose in certain loci before the eutherian-metatherian split, but the mechanisms governing retention of the imprint have diverged in each lineage over the past 150 million years.

## Methods

### Tissue collection

*M. domestica* (gray short-tailed opossum) samples were obtained from the laboratory of Dr. Kathleen Smith, Duke University, under protocols approved by the Institutional Animal Care and Use Committee (IACUC). Liver, brain and kidney tissues from 10 individuals and 10 embryos (belonging to three of the adult females) were dissected and stored at −80°C. DNA was extracted using the Qiagen genomic tip protocol 100/G (Qiagen, Valencia, CA). Total RNA was isolated by homogenization in RNA-Stat60 (Tel-Test, Friendswood, TX) followed by procedures recommended by the manufacturer.

### Analysis of imprint status

The Ensembl genome browser ( http://www.ensembl.org/index.html) was used to identify regions in *M. domestica* orthologous to known imprinted loci in eutherian mammals. Genes were screened for DNA polymorphisms in all 10 adult animals and embryos, and subsequently in the cDNA of heterozygotes. The polymorphism is visibly present in the cDNA sequence if the gene is biallelically expressed, whereas only one allele is visibly present in the sequence if the gene is subject to monoallelic expression. Expression was analyzed in liver, brain and kidney, representing tissues of endodermal, ectodermal and mesodermal origin, respectively. Parental origin of the expressed allele, and thus imprint status, was determined by analyzing heterozygous embryos from mothers homozygous at the corresponding polymorphism.

Primers were designed to amplify regions of exons present in the longest annotated transcript of each gene. PCR was performed using 1.5U of Platinum Taq DNA polymerase (Invitrogen, Carlsbad, CA), 15 pmol of primers, 1.5 mM MgCl_2_ and 10 mM dNTPs in a 25 μl PCR reaction volume with conditions 94°C × 2 min; 94°C × 30 sec, Annealing Temperature × 30 sec, and 72°C × 60 sec for 35 cycles; 72°C × 5 min. The products were purified by gel-extraction and elution in spin columns (Sigma-Aldrich, St. Louis, MO), and then sequenced on an ABI 3130 sequencer (Applied Biosystems, Carlsbad, CA). For heterozygotes, 1 μg RNA from the liver, brain, and kidney was treated with DNase I (Invitrogen), converted to cDNA using Superscript II (Invitrogen) in oligo-dT primed reactions, amplified (PCR being performed using 1.5U of Platinum Taq DNA polymerase, 15 pmol of primers, 1.5 mM MgCl_2_ and 10 mM dNTPs in a 25 μl PCR reaction volume with conditions 94°C × 2 min; 94°C × 30 sec, Annealing Temperature × 30 sec, and 72°C × 60 sec for 35 cycles; 72°C × 5 min, primers detailed in Additional file 1: Table S1) and sequenced on an ABI 3130 sequencer (Applied Biosystems).

*MEST* 3^′^ RACE was performed using the 5^′^/3^′^ RACE kit (Roche Applied Science, Indianapolis, IN). Briefly, first strand synthesis was performed using an oligo dT anchor primer, and the cDNA was further amplified using a forward primer placed in Exon 11 (5^′^ CAGTGAATCCTCATCCAGA 3^′^), since the differences were seen between exon 11 and the 3^′^ end in the wallaby. The products obtained were purified by gel extraction and elution using HiPure columns (Roche Applied Science). Following ligation into the T-easy vector (Promega, Madison, WI) colonies were transformed into JM109 competent cells. These were plated on LB-Agar-Xgal plates (Teknova, Hollister, CA). After overnight incubation at 37°C, white colonies were selected and amplified by whole-cell PCR. T7 and SP6 primers were used for amplification with conditions as described previously [29], and the inserts were sequenced on an ABI 3130 sequencer (Applied Biosystems).

### Analysis of methylation status

With the use of a custom-designed algorithms from Albert Einstein College of Medicine, New York, (Glass, Thompson et al. 2007), CpG-rich domains were identified in genomic regions 100 kb upstream and downstream of the genes selected for investigation in *M. domestica.* Genomic DNA was modified using sodium bisulfite to selectively convert all unmethylated cytosines to uracils, using the Qiagen Epitect Bisulfite Kit (Qiagen). Fourteen CpG-rich regions in the proximity of the chosen genes were then analyzed for their methylation status. Standard and semi-nested PCR assays were designed for bisulfite treated DNA using Primer 3 ( http://frodo.wi.mit.edu/). Converted liver DNA was amplified in PCR reactions, and the products were purified using a QIAquick Gel Extraction Kit (Qiagen). Purified PCR amplicons were cloned using TOPO TA Cloning Kits (Invitrogen) and plasmid DNA was purified using Montage Plasmid MiniprepHTS 96 Kit (Millipore, Billerica, MA). Plasmid DNA was sequenced and the methylation status of the clones was visualized using CpGviewer ( http://dna.leeds.ac.uk/cpgviewer/). We expected an approximately equal proportion of homogeneously methylated and unmethylated clones from regions that function as imprinted DMRs, whereas the clones from CpG-rich regions that are not involved in regulating imprinting are expected to exhibit more heterogeneous methylation profiles.

To determine if methylation of the *M. domestica IGF2R* DMR was allele-specific, we analysed differential methylation in 6 opossum samples (three sets of parent-offspring pairs: the first pair was a father-son pair from which liver tissues were analysed, the other two were mother-embryo pairs). Bisulfite treated DNA was amplified, ligated, transformed, amplified with T7 and SP6 primers and sequenced on an ABI 3130 sequencer (Applied Biosystems), as previously described. Antisense transcription was analyzed in the embryonic cDNA using strand-specific RT-PCR followed by PCR; appropriate primers were positioned immediately upstream and downstream of the DMR.

### Chromatin immunoprecipitation assay

Chromatin was sheared using the Active Motif ChIP-IT Protocol (Active Motif, Carlsbad, CA), modified for the use of tissues instead of cells. Briefly, 50 mg of tissue was crushed using a pestle and mortar, which was submerged in liquid nitrogen. Samples were then immediately incubated for 10 minutes in 10 ml of Fixation solution (1% Formaldehyde in 1X PBS) to cross-link the DNA and associated proteins. The samples were then washed successively in Glycine-stop-fix solution (10X glycine buffer, 10X PBS and dH_2_O) and Cell-scraping solution (10X PBS, dH_2_O, and 100 mM PMSF). The samples were incubated for 30 min in ice-cold lysis buffer (supplemented with 7.5 μl Protease Inhibitor Cocktail, PIC, and 7.5 μl PMSF), and then dounced gently ten times to aid nuclei release. Finally, the samples were resuspended in 330 μl of shearing buffer, and transferred to an ice bath in which sonication was performed. To shear the chromatin into 200 to 1000 bp fragments, nuclei were subjected to sonication five times at 35% amplitude in 10 sec pulses, with a rest of 50 sec between each pulse. Cellular debris was pelleted by centrifugation at maximum speed for 12 min at 4°C. The supernatant containing the sheared DNA was removed and flash-frozen until further use. In order to check the shearing efficiency, 25 μl of the supernatant was incubated with 5 M NaCl and RNase A for 4 hr at 65°C. After further treatment with Proteinase K, phenol-chloroform extraction and ethanol precipitation, the sheared chromatin was visualized on a 2% agarose gel.

The Active Motif ChIP-IT Express Kit (Active Motif) was utilized for immunoprecipitation. ChIP grade antibodies specific for H3K4 dimethylation (H3K4me2) and H3K9 trimethylation (H3K9me3) were obtained from Upstate Cell Signaling Solutions (Millipore). Rabbit serum albumin was also used as a negative control to assess non-specific binding. Ten μl of sheared chromatin was reserved to serve as “input DNA”. The remaining sheared chromatin (6.3 μg) was processed as per manufacturer’s instructions. The input DNA was further purified with RNase A, phenol-chloroform extraction and ethanol precipitation.

All samples were used as template in the PCR reactions to determine the genomic regions enriched for each histone modification. At least three biological replicates were tested for each gene locus. To further assess for allele-specific enrichment, input and immunoprecipitated products were purified, ligated, transformed, amplified using T7 and SP6 primers and sequenced, as described above. The ratio of the two alleles in the input versus the H3K4me2 antibody immunoprecipitated samples was compared. Chi square tests, using Yates correction for small sample size, were used to determine whether the input or the ChIP samples were enriched for a particular allele. A p-value of 0.05 was used as a cut-off for significance.

### Real-time PCR

Relative quantification of the immunoprecipitated DNA was performed with the Applied Biosystems 7900HT Fast Real-Time PCR System (Applied Biosystems). ChIP DNA (2 μl) was utilized in 20 μl reactions, conditions were set as per manufacturer’s instructions (Active Motif). Primers were designed to amplify 150–300 bp products, and were checked before use for efficiency and amplification specificity under standard PCR reaction conditions. SybrGreen Assays were performed to judge the relative enrichment of the different histone modifications relative to the input DNA. The cycle threshold (Ct) value for sample input and immunoprecipitated DNA for each experiment was normalized by subtracting the Ct value for control albumin input and immunoprecipitated DNA, respectively. The enrichment was calculated by subtracting the immunoprecipitated sample’s normalized Ct from the normalized input Ct (Additional file 1: Table S6).

## Results

### Analysis of imprint status

Availability of the genome sequence for *M. domestica* enabled us to use the Ensembl genome browser to bioinformatically search for orthologues of Eutherian imprinted genes. We focused on six known imprinted genes in mouse that also had a high level of genomic sequence conservation in eutherians, namely *MEST*[8,30], *COPG2*[31], *HTR2A*[32], *L3MBTL*[33], *IMPACT*[34,35] and *PLAGL1*[36]*.*

**Figure 1:**
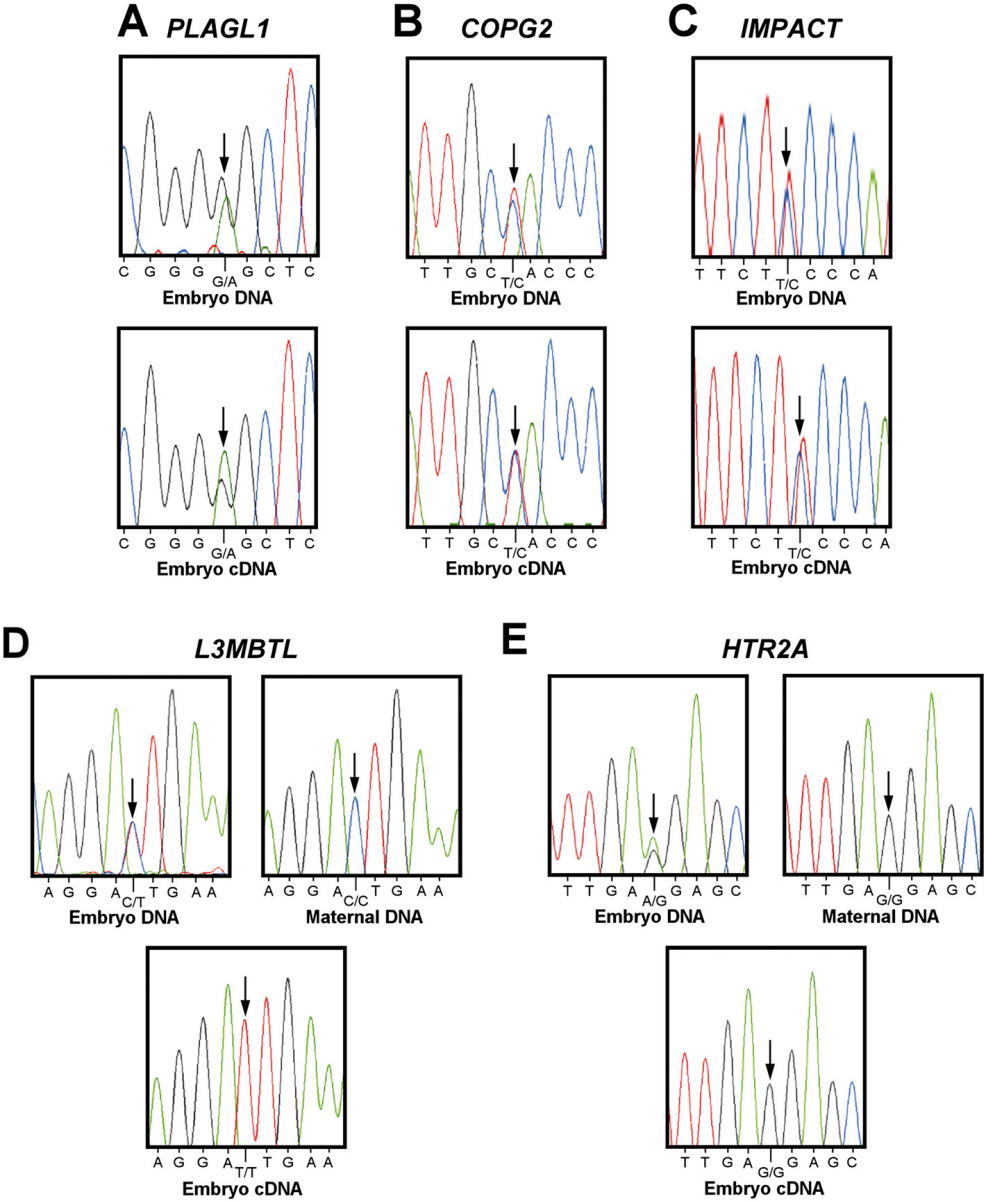
**Imprinting analysis of metatherian orthologues of eutherian imprinted genes in *M. domestica.*** The five gray short-tailed opossum genes investigated are *(****A****) PLAGL1*, *(****B****) COPG2*, *(****C****)* IMPACT, *(****D****) L3MBTL* and *(****E****) HTR2A*. The arrow denotes the polymorphism used to determine the imprint status of the gene. *PLAGL1* (n = 2), *COPG2* (n = 2), and *IMPACT* (n = 2) are not imprinted since the polymorphism is present in both the genomic DNA and the cDNA. In contrast, *L3MBTL* (n = 4) and *HTR2A* (n = 3) are imprinted since the genomic polymorphism is absent in the cDNA, demonstrating that only one allele is expressed. Using those samples where the offspring are heterozygous and the mothers are homozygous at the same polymorphic site, it was determined that *L3MBTL* and *HTR2A* were expressed from the paternal and maternal alleles, respectively.

**Figure 2:**
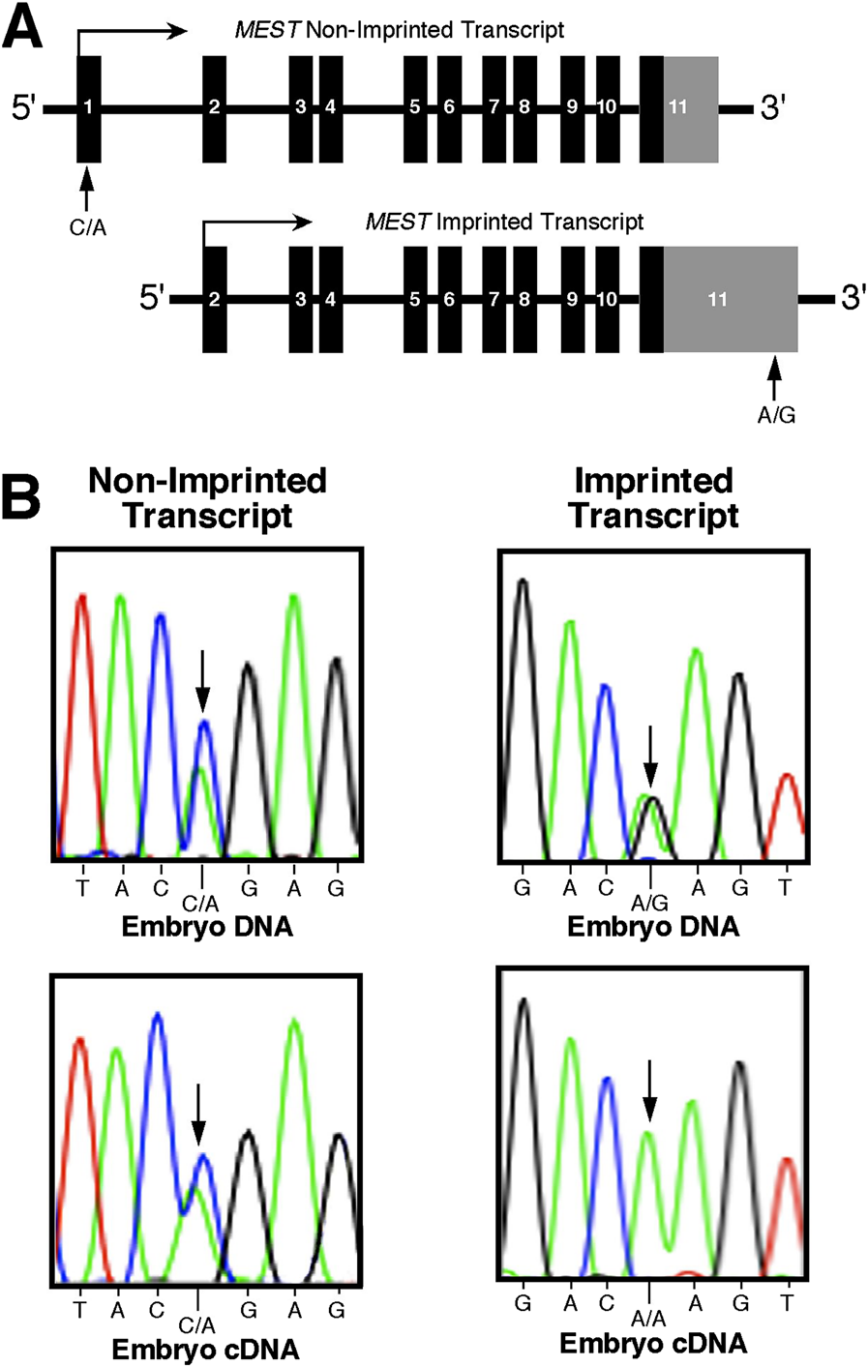
**Analysis of imprinting at the *MEST *locus in *M. domestica.**** (****A****)* 3′ RACE distinguished two alternative *MEST* transcripts. The vertical arrows indicate the polymorphic sites used to analyze the imprint status of the two transcripts. *(****B****)* Transcript 1 (n = 2) is imprinted since the genomic A/G polymorphism is absent in the cDNA, demonstrating that only one allele is expressed. The parental origin of expression for transcript 1 could not be determined because a heterozygous offspring with a mother homozygous at the A/G polymorphic site was not found. In contrast, transcript 2 (n = 1) is not imprinted since the C/A polymorphism is present in both the genomic DNA and the cDNA.

*PLAGL1, COPG2,* and *IMPACT* showed biallelic (non-imprinted) expression in 2 amongst the 10 *M. domestica* adults investigated (Figures 1A, 1B and 1C, Table 1, Additional file 1: Table S1). The bi-allelic expression pattern was consistent in liver, brain and kidney tissues. In contrast, *L3MBTL* was expressed exclusively from the paternal allele, as judged from 2 different SNPS in 4 heterozygotes amongst the 10 adults and 10 embryos tested (Figure 1D, Table 1, Additional file 1: Table S1). Similarly *HTR2A* was expressed from only the maternal allele (Figure 1E, Table 1, Additional file 1: Table S1) in 3 heterozygotes containing two different SNPs amongst the 20 individuals investigated. Two *MEST* transcripts are present in eutherians and also in the metatherian tammar wallaby [8]. 3' RACE (Rapid Amplification of cDNA Ends) likewise revealed two transcripts in the gray short-tailed opossum. Utilizing SNPs in exon 1 and the 3' UTR (Figure 2A), transcript 1 was shown to be monoallelicaly expressed in one individual, whereas transcript 2 was expressed from both alleles in one individual (Figure 2B). For all the genes determined to be imprinted in *M. domestica*, monoallelic expression was observed in all three tissues examined (liver, brain and kidney), both during early development and in adulthood (data not shown).

**Table 1:** **Genes analyzed for imprint status in *M. domestica***

**Gene**	**Function**	**Imprint status**		
		**Human**	**Mouse**	**Opossum**
***COPG2***	Binds to CDC42	Paternal	Maternal	Biallelic
***L3MBTL***	Polycomb protein	Paternal	Unknown	Paternal
***IMPACT***	Needed for cell growth	Biallelic	Paternal	Biallelic
***HTR2A***	Serotonin receptor	Maternal	Maternal	Maternal
***PLAGL1***	Transcription factor	Paternal	Paternal	Biallelic
***MEST***	Member of the alpha/beta hydrolase fold family	Transcript specific paternal expression	Transcript specific paternal expression	Transcript specific monoallelic expression*

*Unable to determine the parental origin of the expressed allele.

### Analysis of differential methylation

**Figure 3:**
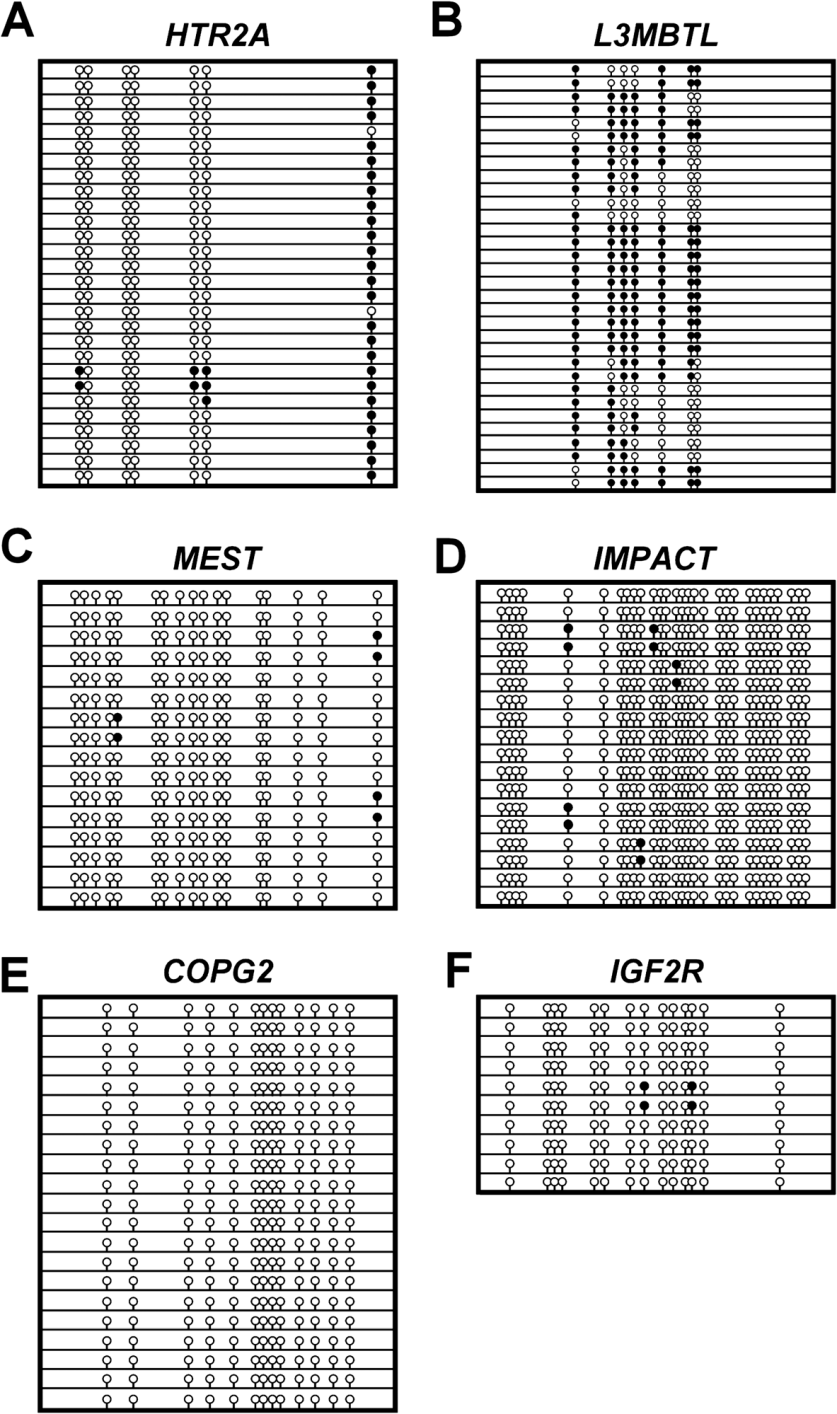
**Methylation profiles of *M. domestica *CpG-rich regions close to orthologues of eutherian imprinted genes.** Bisulfite-modified genomic DNA was PCR amplified and cloned. Each line denotes an individual cloned allele. Representative CpG-rich regions analyzed are *(****A****)* putative promoter region of *HTR2A*, *(****B****)* 5^′^ upstream region of *L3MBTL*, *(****C****)* 5^′^ upstream region of *MEST, (****D****)* 5^′^ upstream region of *IMPACT*, *(****E****)* 5^′^ upstream region of *COPG2*, and *(****F****)* putative promoter region of *IGF2R*. Unfilled circles depict unmethylated cytosines at CpG sites, while filled circles depict methylated cytosines. The genomic co-ordinates of these regions are listed in Additional file 1: Table S3.

Twelve CpG-rich regions were identified that were within 100 kb distance of the six genes selected for imprinting analysis in *M. domestica.* These were analyzed for their methylation status in the liver DNA of two individuals. Surprisingly, the CpG-rich regions of the imprinted genes identified in *M. domestica* were not differentially methylated but rather exhibited a hypomethylated state, or in the case of *L3MBTL* a relatively hypermethylated state (Figures 3A, 3B, and 3C, Table 2, Additional file 1: Table S2 and Table S3). As expected, the CpG-rich regions investigated for the biallelically expressed genes were also not differentially methylated but rather were hypomethylated (Figure 3D and 3E, Table 2, Additional file 1: Table S2 and Table S3).

**Table 2:** **Methylation status of CpG islands in vicinity of *M. domestica *orthologues of eutherian imprinted genes**

**Gene**	**CpG-rich region location**	**Conservation in mouse**	**DMR**
***PLAGL1***	Putative promoter	Yes	No
***L3MBTL***	Putative promoter, Intron 2, Intron 4	Yes	No
***IMPACT***	20 kb upstream of promoter, Putative promoter	Yes	No
***HTR2A***	10 kb upstream of promoter, Putative promoter	Yes	No
***COPG2***	4 kb upstream of promoter	Yes	No
***MEST***	4 kb upstream of promoter	Yes	No
***IGF2R***	38 kb upstream of promoter	Yes	No
	Intron 11	No	Yes

**Figure 4:**
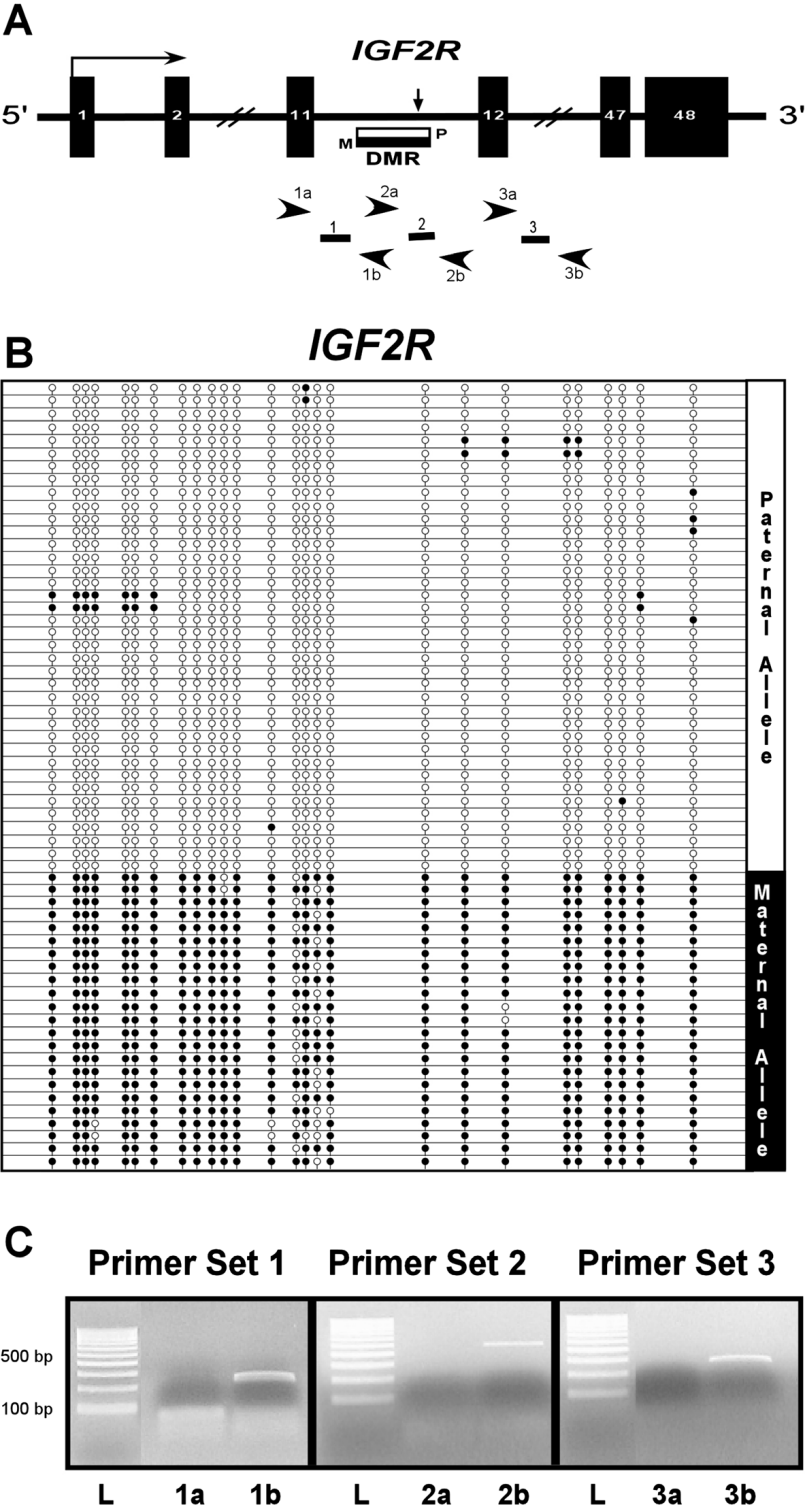
**Methylation profile and antisense transcript analysis for the novel DMR identified in intron 11 of *M. domestica IGF2R.****(****A****) IGF2R* is represented graphically. The three sets of primers used in strand-specific RT-PCR analysis are labeled as 1, 2 and 3. The reverse transcription primers for anti-sense strand detection are labeled as 1a, 2a, and 3a while those for sense-strand detection are labeled as 1b, 2b, and 3b. *(****B****)* Parent-of-origin analysis was performed at the DMR by analyzing methylation of cloned alleles for an individual bearing an (A/T) SNP at chr2: 442,443,695 with a mother homozygous for A at the same location. Only the allele inherited from the mother is methylated. *(****C****)* Strand-specific RT-PCR analysis using primers described above shows a PCR product only by reverse transcription utilizing 1b, 2b band 3b; L represents the 100 bp DNA ladder. This demonstrates that the gene is expressed at this locus but there is no associated anti-sense transcript.

We previously showed that imprinting at the *IGF2R* locus in the opossum *(Didelphis virginiana)* occurs in the absence of the DMRs required for maternal expression in the mouse [37]. Thus, additional CpG-rich regions in *IGF2R* of *M. domestica* were screened for differential methylation. A CpG-rich region located 38 kb upstream of the putative transcription start site of *IGF2R* and another within intron 11 were identified and examined. The region upstream of *IGF2R* was largely hypomethylated (Figure 3F, Table 2, Additional file 1: Table S2 and Table S3). In contrast, the region within intron 11 exhibited differential methylation (Figures 4A and 4B). This DMR appears to be absent in eutherians, since the sequence is not conserved in the mouse, dog, and human *IGF2R*. An A/T polymorphism in the liver and embryonic DNA of two individuals (out of the six analyzed) helped establish the fact that each of the alleles was either completely methylated or completely unmethylated (data shown for the embryo in Figure 4B). Further analysis of the heterozygous embryo with a homozygous mother showed that only the maternal allele of *IGF2R* was expressed in the embryo, as previously reported in *D. virginiana*[6]. Moreover, the maternal (expressed) allele was mostly methylated, whereas the paternal (non-expressed) allele was mostly unmethylated. This is similar to the methylation pattern of the intron 2 DMR in the mouse, which leads to production of the antisense transcript *Air* from the paternal allele [37]. An antisense transcript originating from this region was however not detected. The antisense strand-specific RT primer around the DMR failed to produce any product while the sense strand-specific primer was used as a positive control to show that the gene itself was expressed in this region (Figure 4A and 4C, Additional file 1: Table S4).

### Analysis of histone modifications at the promoter region

Chromatin immunoprecipitation was performed on 6 adult opossum tissues (4 liver, 1 brain, 1 kidney) using antibodies specific for H3K4me2 and H3K9me3 modifications. Control assays for the promoter region of the highly expressed albumin gene (*ALB*) showed enrichment for the activating H3K4me2 mark but not for the repressive H3K9me3 mark as compared to *ALB* introns and exons in *M. domestica* liver tissue. Similarly, in brain tissues, the promoter of the presumed non-expressed *MHC* gene was enriched for the repressive H3K9me3 mark.

**Figure 5:**
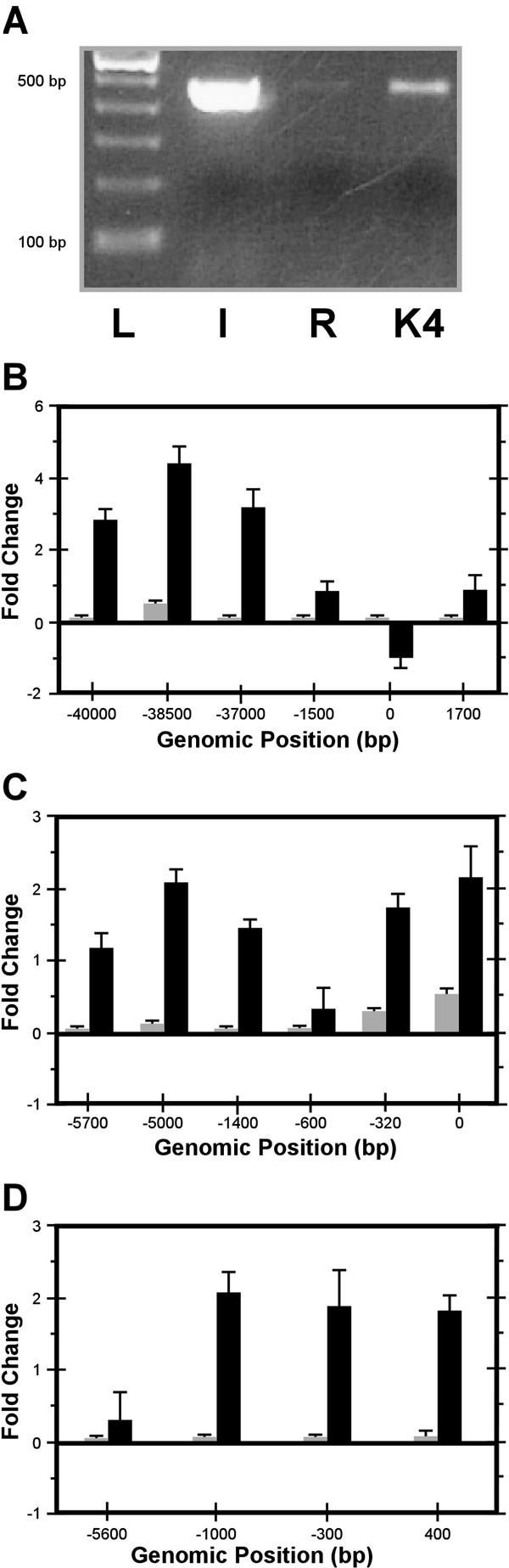
**Analysis of histone modifications at the promoter regions of *M. domestica *orthologues of eutherian imprinted genes.***(****A****)* Chromatin immunoprecipitation with H3K4me2 antibody followed by PCR amplification is shown for the *IGF2R* promoter region; L denotes the 100 bp ladder, I the input, R the sample immunoprecipitated with rabbit serum albumin (i.e. non-specific pull-down), and K4 the sample immunoprecipitated with H3K4me2 antibody. Analysis of enrichment was performed by real-time PCR following chromatin immunoprecipitation with H3K4me2 and H3K9me3 antibodies at the promoter regions of *(****B****) IGF2R*, *(****C****) HTR2A,* and *(****D****) L3MBTL*. The fold change is plotted on the Y-axis, and the distance from the putative transcription start site (0) on the X-axis. The black bars represent H3K4me2, and the grey bars represent H3K9me3.

The regions upstream of *L3MBTL*, *HTR2A* and *IGF2R* were enriched for the active H3K4me2 mark but not for the repressive H3K9me3 mark (Figure 5, Table 3, Additional file 1: Table S5 and S6). To assess whether the active modification was associated with only one allele, ChIP was performed on individuals polymorphic in these regions. Clones of the immunoprecipitated samples as well as input DNA were analyzed, revealing that in the case of *IGF2R* and *HTR2A*, H3K4me2 was significantly enriched on one allele (Table 3), while the input contained equal representation of both alleles. In the case of *L3MBTL*, although the H3K4me2 enrichment on one allele was not significant using the standard p-value of 0.05, the enriched allele was established as the paternal allele (since the polymorphic individual’s father was homozygous at this locus). This is a strong indication that H3K4me2 is associated only with the expressed paternal allele.

**Table 3:** **Allele-specific enrichment of histone modification H3K4me2 in promoter regions of *M. domestica *orthologues of eutherian imprinted genes**

**Gene**	**Polymorphism**	**Genomic location**	**Input: Number of clones**		**H3K4 dimethyl ChIP sample: Number of clones**		**p-Value for Allele-specific enrichment**	
			**Allele 1**	**Allele 2**	**Allele 1**	**Allele 2**	**Input**	**H3K4 ChIP sample**
***L3MBTL***	C/A	1: 315,862,133*	7	11	18	9	0.346	0.08
***HTR2A***	G/C	4: 322,101,216	8	7	4	11	0.715	0.02
***IGF2R***	T/C	2: 445,407,861	5	6	1	10	0.668	0.006

*Genomic location obtained from MonDom4 Version of the genomic sequence for *M. domestica*.

## Discussion

In this study, we determined the imprint status of *M. domestica* orthologues of genes imprinted in eutherian mammals. Genes found to be imprinted in *M. domestica* were screened for differential methylation and histone modifications, epigenetic modifications that are known to play a critical role in the establishment and regulation of imprinted gene expression in eutherians. Three of the six genes analyzed (*L3MBTL*, *HTR2A* and *MEST*) exhibited monoallelic expression in this metatherian, showing conservation of imprint status with eutherians. Interestingly, *MEST* had one monoallelic and one biallelic transcript, which is consistent with reports from mice, humans and the tammar wallaby [8,30]. Furthermore, parent-of-origin expression analysis showed that *L3MBTL* was expressed from the paternal allele, while *HTR2A* was expressed from the maternal allele, also consistent with mouse and human parental expression patterns (Table 1). Since two SNPs each were used in the analysis of parent-of-origin expression for both genes, we ruled out genetic heterogeneity as being the cause of monoallelic expression. *MEST* parent-of-origin expression in *M. domestica* remains undetermined since no informative polymorphisms in parent-offspring duos were found; however, due to the high conservation of imprinting expression at this locus, it is likely that the gene is paternally expressed, as it is in another metatherian, the tammar wallaby [8].

Although metatherian imprinted loci share many homologous features with eutherians, differences are also apparent. In contrast to eutherians, monoallelic expression of *L3MBTL*, *HTR2A* and *MEST* is maintained in tissues derived from the three germ layers (liver, brain and kidney), whereas polymorphic imprinting is common at these loci in eutherians [1]. Additionally, only a subset of genes imprinted in mice and humans appear to be imprinted in metatherians, even though the genes themselves are phylogenetically conserved. Interestingly, even genes lying within conserved eutherian “imprinted domains” can lack coordinate imprinting in the opossum. For example, in *M. domestica, MEST* exhibits imprinted expression while the adjacent *COPG2* is expressed from both alleles. In mice, however, both genes are imprinted. Interestingly, *COPG2* is also biallelically expressed in cattle [38] which suggests that imprinting of this gene may not have evolved until well after the metatherian-eutherian divergence. Similarly, in the tammar wallaby, *UBE3A* and *SNRPN* (the only two genes of the orthologous Prader Willi Syndrome - Angelman Syndrome domain that could be identified) are located on different chromosomes and are not imprinted [39]. Unlike humans and mice which show imprinted expression of the retrotransposed gene *PEG10* and neighboring *SGCE*, imprinted expression is restricted to *PEG10* in the tammar wallaby [7].

Imprint regulatory features also differ between the two mammalian lineages. Out of the 14 CpG-rich regions analyzed in *M. domestica*, a number of which are known to serve as ICRs for the adjacent genes in eutherians, only one DMR was identified – a region within intron 11 of *IGF2R*. Moreover, this DMR is not conserved in eutherians. Identification of this DMR demonstrates the possibility that such regions may have been missed in previous analyses in metatherians due to incomplete sequence availability. Future studies need to focus on the deterministic role and germ cell origin for this DMR in order to elucidate its function, if any, in governing *IGF2R* imprinting in metatherians. Given that the maternal allele is methylated, it is possible that this mark is a maternally-derived gametic imprint. Study of gametes will be required to determine if this region indeed represents a gametic imprint mark. Since we have been able to confirm the maternal allele-specific methylation only in one embryo due to limited availability of parent-offspring matched samples, further studies would be needed to confirm the methylation pattern in many more animals and tissues. Although we were unable to detect an anti-sense transcript from this region in embryonic tissue, the possibility remains that such a transcript is produced in the germ-line or in a developmental-stage specific manner in *M. domestica*. Nonetheless, the lack of conservation of the *IGF2R* intron 11 DMR in the human, dog and mouse indicates that DMRs are not always conserved between eutherians and metatherians. Other DMRs, such as the *PEG10* DMR, also vary between the two Therian infraclasses. In the tammar wallaby, the *PEG10* DMR is limited to its promoter region, while in eutherians the maternal allele-specific methylation spreads to the promoter of the adjacent gene, *SGCE*, leading to expression from the paternal allele [7]. Somatic DMRs are also not always conserved between eutherian species, for example, *Impact* is imprinted in mice and has an intronic DMR but its human counterpart lacks this DMR and exhibits biallelic expression [34]. In *M. domestica, IMPACT* shows no evidence of a DMR in its intronic region, similar to its status in humans. Interestingly, the reported DMR sequence in *IGF2* of *M. domestica* bears 50% similarity to humans [18], yet its demethylation leads to activation of the maternal allele, instead of silencing the paternal allele as seen in mice.

Histone modifications at the promoters of imprinted genes also vary between eutherians and *M. domestica*. The presence of the allele-specific activating mark H3K4me2 at the putative promoters of *IGF2R*, *HTR2A* and *L3MBTL* is consistent with a role of histones in marking the imprinted loci in metatherians. However, unlike eutherians, the repressive mark H3K9me3 is absent from the promoters of imprinted genes in *M. domestica*. Nevertheless, this is the first study in metatherians that demonstrates the presence of histone modifications at imprinted loci and suggests that they could serve as the “primordial imprint mark” in the absence of differential methylation. The presence/absence of other histone modifications as well as the germ-line derivation of these marks remains to be elucidated. Moreover, experiments showing abolishment of imprinting from disruption of these histone tail modifications will be necessary to determine their role in imprinting, and to prove that their presence is not just a mere consequence of imprinted expression.

## Conclusions

Based upon the differences in imprinted gene repertoires and the regulatory mechanisms of imprint control between metatherians and eutherians, it appears that the features of genomic imprinting observed in metatherians are not exactly the same as imprinting in the eutherian lineage. In metatherians, genomic imprinting is conserved at only a subset of eutherian imprinted genes, suggesting that this phenomenon originated before the eutherian-metatherian split, but some genes were specifically imprinted much later [40,41]. It would be interesting to explore whether there are metatherian-specific imprinted loci to corroborate this postulate. The *H19* non-coding RNA is present in the tammar wallaby, and was shown to be maternally expressed and associated with a differentially methylated region in the *IGF2* imprinted domain [42]. To date, this is the first and only locus where all the regulatory features of imprinting appear to be conserved between eutherians and metatherians. Thus, Smits et al. suggest sequential evolution of imprinting between the two lineages. However, they also suggest that imprinting of “singleton imprinted genes” such as *MEST*, *IGF2R* and *PEG10* evolved at separate times in metatherians and eutherian mammals. Suzuki et al. suggest that certain imprinted domains such as *KCNQ1* may have evolved specifically in eutherians once placentation patterns became more complex [43].

Our results show that while there are similarities in imprinted genes between metatherians and eutherians, stark differences also exist. It is imperative to extend the evolutionary analyses of imprint control mechanisms to many more regions before we can conclusively determine whether imprinted genes evolved in a convergent or divergent manner, or possibly both. Identification of *IGF2R* intron 11 DMR highlights the need for unbiased methylation analyses in and around metatherian imprinted genes. The complete repertoire of histone modification marks and their role in regulating genomic imprinting in metatherians also remains to be investigated. Once these genome-wide epigenetic marks are equally well defined in metatherians and eutherians we can make strong conclusions regarding the evolution of genomic imprinting in placental mammals.

## Abbreviations

DMR: Differentially methylated region; H3K4me2: Histone3 Lysine4 dimethylation; H3K9me3: Histone3 Lysine9 trimethylation.

## Competing interests

The authors declare that they have no competing interests.

## Authors’ contributions

RDC performed experiments for the imprinting analysis, antisense for *IGF2R* and histone modifications as well as wrote the manuscript. NA did the experiments for CpG island analysis. MIK analyzed the *IGF2R* DMR. JRW, TSM, MK, KLT and JMG analyzed the *M. domestica* genome and chose the genes and CpG islands for analysis of imprinting patterns. SKM and RLJ advised on all stages of the work. All authors read and approved the final manuscript.

## Supplementary Material

Additional file 1 **Table S1.** Primers and genomic coordinates for SNPs used to analyze imprint status in M. domestica. **Table S2.** (**A**) Forward primers used for methylation analysis. (**B**) Reverse primers used for methylation analysis. **Table S3.** Genomic coordinates of regions analyzed for methylation in *M. domestica.* Table S4. Primers used for *IGF2R* antisense detection. **Table S5.** Primers used for the analysis of histone modifications. **Table S6.** Relative enrichment of histone modifications at *M. domestica* imprinted loci. Click here for file
