# Supplementary material for: Convergent and divergent evolution of genomic imprinting in the marsupial Monodelphis domestica
Source: BMC Genomics. 2012 Aug 16;13:394. doi: 10.1186/1471-2164-13-394 (PMC3507640; doi:10.1186/1471-2164-13-394)
Supplement: Additional file 1 — Table S1. Primers and genomic coordinates for SNPs used to analyze imprint status in M. domestica. Table S2. (A) Forward primers used for methylation analysis. (B) Reverse primers used for methylation analysis. Table S3. Genomic coordinates of regions analyzed for methylation in M. domestica. Table S4. Primers used for IGF2R antisense detection. Table S5. Primers used for the analysis of histone modifications. Table S6. Relative enrichment of histone modifications at M. domestica imprinted loci. [file 1471-2164-13-394-S1.doc]

**Supplementary Table 1:** Primers and genomic coordinates for SNPs used to analyze imprint status in *M. domestica*

| **Gene** | **Forward Primer** | **Reverse Primer** | **SNP Location**  **Chromosome: Coordinate** |
| --- | --- | --- | --- |
| *L3MBTL* | GAGACTAATCACCTCACCTCCT  AGTCCTCAGCCAGCCAG | CCGAGAAGAGGGAGCC  GAGAAGAATATAGGGTGAGAGTGA | 1: 385,955,543*  1: 385,957,311 |
| *HTR2A* | GTGAGTATCATTTGTGTTCCCT  GAGACACCCAGAACTGAACAC | ACTGAGTGACAGAGATAAAGCTG  GCATCTGGAGGGCTTC | 4: 322,102, 343  4: 322,105,260 |
| *MEST* | GGATCCCCTGTGTGGCTC  CTCCACATTCTCTTCCAATAG | TCGTCCCCAGCCTCAC  ACAGAGCAAGGAAAACGAC | 8: 190,443,889  8: 190,463,734 |
| *PLAGL1* | CATGAAATGATATTGTGTGGTTC | CAGACACCTAGCGAGAAAGG | 2: 422,112,031 |
| *COPG2* | GTGGCCATGAAACACC | AAAGCAAACAGGACAAGTATTAC | 8: 190,486,069 |
| *IMPACT* | AGAGACCGACTGTGGC | CTGTAAGCAGAGTGTCCAT | 3: 260,030, 960 |
| *IGF2R* | TTCTACAGATCATAAATTAAGC | CTTTAATCATTTCCTCCC | 2: 442,443,695 |

*Genomic location obtained from MonDom4 Version of the genomic sequence for *M. domestica.*

**Supplementary Table 2A:** Forward primers used for methylation analysis.

| **Gene Product** | **Forward Primer 1** | **Forward Primer 2** |
| --- | --- | --- |
| ***COPG2.3*** | GTTATTAGGAGGGTTAGGGTTTAGT | None |
| ***COPG2_As1*** | ATTAATGGTTTTAAGTTTTGGGTATGG | None |
| ***COPG2_As2*** | GGTTTTAAGTATTAGGGGTTTTAAGAG | None |
| ***HTR2A.1*** | GGTTGGAGAAGTTGTATATTTATGT | None |
| ***HTR2A.2*** | GTAAGGTAGAAGAGAAGAAGTAAGTT | None |
| ***IGF2R_A.3*** | GTGGGAAGTTGAGGGTTTTT | None |
| ***IGF2R_A_As1*** | TGGTTATAGGGATAAGGTTAGGTAAAA | None |
| ***IGF2R_A_As2*** | GGAAGTTTATGGATTAGGGGTTATTAG | None |
| ***IGF2R_A_As4*** | TTTAGGAGTTGTGTAGGTAAGGAAGTG | None |
| ***IGF2R_B.1*** | GTTTATTTGTATATGGAAAGGAGAA | None |
| ***IGF2R_B.2*** | GTTTTTTTGGATTTTGAAGATTTGT | None |
| ***IGF2R_B_As1*** | GGATTATTTTTAGGGAGGAAATGATTA | TGGAAGAATTTTATGGTATTTTTGAAG |
| ***IMPACT_A.1*** | TAAGATTAGATTGGATTTTAAGTTG | None |
| ***IMPACT_A.2*** | AGTTAGAGAGGGTTGATATTTGTTT | None |
| ***IMPACT_A.3*** | ATGTGATTAGTTTTATTTTGGATTT | None |
| ***IMPACT_B.2*** | AAAAGTAGAGGTGGAGGGTTGTATAG | None |
| ***IMPACT_B_As1*** | AGTTGTATTTAAAAGTGTGTTTTAGGATT | TTTATTTGTGGTAGTTGATATAAATTTTG |
| ***L3MBTL_A_As1*** | ATGTAGGTTAGAGAGGGGTTTTGTTAT | None |
| ***L3MBTL_B_As1*** | AATAATTAGGTTTATTTGGAGGGAATG | TTATGGGTTTATTAGTTATGGGTTGAG |
| ***L3MBTL_B_As2*** | GATTGATTCGTAGGTTGTTTTTAGGTA | None |
| ***L3MBTL_C.1*** | TTTTATAGTTTTTGTAATGGGAGTT | None |
| ***L3MBTL_C_As1*** | TGATTAGGTTAGGGATTTTAGGAGTTT | GTAGAGATGGGGAGATTTTAGAGGTAG |
| ***L3MBTL_C_As2*** | ATTTATGGGTTATTGAAGGGTTTTATT | None |
| ***MEST_A_As2*** | TGTAGTTTAATGGTTGTTAGGGGTAAT | None |
| ***MESTB_As1*** | TTTTGTATATAATGAGGTAAAATTTGTGAA | None |
| ***MESTB_As2*** | GGTTGGGGTATGGATAGTTTTGG | None |
| ***MethControl.2*** | TAAAAGGATTGGAAGTTATGTTATTGG | ATATTTATAATTGGAGGGTTGGAGAAA |
| ***PLAGL1_As1*** | AGTAGTGTTGTTAATAAATTGAAGGATTT | GAAAGTATGTTTTTATTGGTTTTTGGA |
| ***PLAGL1_As2*** | TGTTGGGTTGTTGTTTTATTTAGGTAT | None |
| ***Unmeth_control_As1*** | GGGAAGGGAAGTGTTTAGTTTTT | None |
| ***Unmeth_control_As2*** | GGAAGGAGTTTAGAGTAGTTTTTAGTTGG | None |

**Supplementary Table 2B:** Reverse primers used for methylation analysis.

| **Gene Product** | **Reverse Primer 1** | **Reverse Primer 2** |
| --- | --- | --- |
| ***COPG2.3*** | CTAAAAATAACCTAATTTCCAAAAA | None |
| ***COPG2_As1*** | CTCTTAAAACCCCTAATACTTAAAACC | None |
| ***COPG2_As2*** | AACTAAAATCCCTCCTCCTCCACTAAT | None |
| ***DIO3.1*** | AAAAATAACTTAACGAAACCCTAA | None |
| ***HTR2A.1*** | AACTTACTTCTTCTCTTCTACCTTAC | None |
| ***HTR2A.2*** | AATTAACCACACTCGAAATACTAAT | None |
| ***IGF2R_A.3*** | ACATCTTCCTTCTCCAAACTTAC | None |
| ***IGF2R_A_As1*** | CTAATAACCCCTAATCCATAAACTTCC | None |
| ***IGF2R_A_As2*** | CTTCTCCTCCCCACTTCTCTATC | None |
| ***IGF2R_A_As4*** | CCCACTCCAAACCAAATAAAAA | None |
| ***IGF2R_B.1*** | ACAAATCTTCAAAATCCAAAAA | None |
| ***IGF2R_B.2*** | CTTCAAAAATACCATAAAATTCTTC | None |
| ***IGF2R_B_As1*** | CAATCTCTACCATTCCCTCTAATTCTA | None |
| ***IMPACT_A.1*** | TCATTCTAAAAAAAATCCTCCATAC | None |
| ***IMPACT_A.2*** | AAATCCAAAATAAAACTAATCACAT | None |
| ***IMPACT_A.3*** | AAACAAAATACCTCTTAATCAAAAA | None |
| ***IMPACT_B.2*** | CAACATTCTCTCCACTTAAAAACTC | None |
| ***IMPACT_B_As1*** | AAAACTATACAACCCTCCACCTCTACT | None |
| ***L3MBTL_A_As1*** | AAATTCCCTTTTCCTAAAATCCTTTAT | ATTCCCCTAAACTTAAATCAACATTTC |
| ***L3MBTL_B_As1*** | TACCTAAAAACAACCTACGAATCAATC | None |
| ***L3MBTL_B_As2*** | AACCCTAACCTAAAATAACCAAACTCC | AAACTCCTAAAATCCCTAACCTAATCA |
| ***L3MBTL_C.1*** | TTCTTACATCCCTAACTCTACCTAA | None |
| ***L3MBTL_C_As1*** | AATAAAACCCTTCAATAACCCATAAAT | None |
| ***L3MBTL_C_As2*** | CAAATCATTTAAACTCCACAATAACCT | CTTTACCTCCTTTCCTAATCCTTAAAC |
| ***MEST_A_As2*** | ACCTTATATTATCCATTTCCCAAAACT | CTAAAAACACTCCCCACTCCATTAC |
| ***MESTB_As1*** | CCAAAACTATCCATACCCCAACC | None |
| ***MESTB_As2*** | CAAATAAACAAAAACAACCAATAAACA | None |
| ***MethControl.2*** | TTACAATAACATTTACAATAACCTACATCA | None |
| ***PLAGL1_As1*** | ATACCTAAATAAAACAACAACCCAACA | None |
| ***PLAGL1_As2*** | TTTCTTTTCTCTCTACAAACAAAATCT | AACTATCAAACACCAACCAAAAATTAC |
| ***Unmeth_control_As1*** | CCCAACTAAAAACTACTCTAAACTCCTT | None |
| ***Unmeth_control_As2*** | AACCTAAAACTTCTCTACCCCTAAAAA | None |

**Supplementary Table 3:**  Genomic coordinates of regions analyzed for methylation in *M. domestica*

| **Gene Product** | **Genomic Start** | **Finish** | **Size (bp)** |
| --- | --- | --- | --- |
| ***COPG2.3*** | 186437881* | 186438252 | 371 |
| ***COPG2_As1*** | 186436617 | 186436874 | 257 |
| ***COPG2_As2*** | 186436847 | 186437315 | 468 |
| ***HTR2A.1*** | 312085321 | 312085795 | 474 |
| ***HTR2A.2*** | 312085769 | 312086146 | 377 |
| ***IGF2R_A.3*** | 442406608 | 442407048 | 440 |
| ***IGF2R_A_As1*** | 442405350 | 442405645 | 295 |
| ***IGF2R_A_As2*** | 442405618 | 442405948 | 330 |
| ***IGF2R_A_As4*** | 442406351 | 442406648 | 297 |
| ***IGF2R_B.1*** | 442481958 | 442482459 | 501 |
| ***IGF2R_B.2*** | 442482434 | 442482929 | 495 |
| ***IGF2R_B_As1*** | 442482902 | 442483403 | 501 |
| ***IMPACT_A.1*** | 260054981 | 260055316 | 335 |
| ***IMPACT_A.2*** | 260055322 | 260055856 | 534 |
| ***IMPACT_A.3*** | 260055831 | 260056390 | 559 |
| ***IMPACT_B.2*** | 260031046 | 260031493 | 447 |
| ***IMPACT_B_As1*** | 260030640 | 260031076 | 436 |
| ***L3MBTL_A_As1*** | 385955382 | 385955901 | 519 |
| ***L3MBTL_B_As1*** | 385956549 | 385956980 | 431 |
| ***L3MBTL_B_As2*** | 385956953 | 385957398 | 445 |
| ***L3MBTL_C.1*** | 385957265 | 385957551 | 286 |
| ***L3MBTL_C_As1*** | 385957384 | 385957831 | 447 |
| ***L3MBTL_C_As2*** | 385957804 | 385958110 | 306 |
| ***MEST_A_As2*** | 186358831 | 186359320 | 489 |
| ***MESTB_As1*** | 186360936 | 186361329 | 393 |
| ***MESTB_As2*** | 186361307 | 186361718 | 411 |
| ***MethControl.2*** | 126702427 | 126702820 | 393 |
| ***PLAGL1_As1*** | 422128619 | 422129116 | 497 |
| ***PLAGL1_As2*** | 422129089 | 422129421 | 332 |
| ***Unmeth_control_As1*** | 103840827 | 103841343 | 516 |
| ***Unmeth_control_As2*** | 103841314 | 103841552 | 238 |

*Genomic location obtained from MonDom4 Version of the genomic sequence of *M. domestica.*

**Supplementary Table 4:** Primers used for *IGF2R* antisense detection.

| **Strand-specific RT-primer** | **Forward Primer** | **Reverse Primer** | **Product Size (bp)** |
| --- | --- | --- | --- |
| 1) 1a: CATCAATATCTGTCACAAAG  1b: AACGCTTAGATTTGGCTT | GGTGAGTATAACATCCAGAGAA | GCCTCAGTCTAAGAAGTTCTC | 180 |
| 2) 2a: gatgttgtcagaaggttgt  2b: GTCGGTTTCAACAATCC | ATGTGATACACCGTAGTCG | ATGAGTATGTGTTTACATGTACG | 476 |
| 3) 3a: CTAGGCAAGTTTGTGTCTTC  3b: AACCTTTTATTCATCCTGTT | GTGTCAAGTATTTGAAGGTAGGA | GCTTTAAGACACATCAAAAGGAA | 242 |

**Supplementary Table 5:** Primers used for the analysis of histone modifications.

| **Gene Product** | **Forward Primer** | **Reverse Primer** | **Distance from Transcription Start Site (bp)** |
| --- | --- | --- | --- |
| ***HTR2Ap6*** | GACATTACCTAGTCCACTCTCCT | AGAAGCTGAGGCAAACAGA | -2600 |
| ***HTR2Ap3*** | CAAATTACCATCTGATTGTGTG | TGAAAACTTACATGAGGCTTCA | -1000 |
| ***HTR2Ap1*** | TGTGGGTGACACTGGATA | ACCTATTTTACTCAGCATGTGT | -300 |
| ***HTR2Ain1*** | GTGAGTATCATTTGTGTTCCCT | ACTGAGTGACAGAGATAAAGCTG | +400 |
| ***L3MBTLp6*** | CCTTATCTTCCATCCAAGAATC | AGGTCCTGAGTTCAAATTTGAC | -2700 |
| ***L3MBTLp5*** | GATGAAGGTCACACAACCA | GCTGCATGACAAGGAAAG | -2000 |
| ***L3MBTLp4*** | CCATTTGCCTCCTATGTCTT | ATTTGTACATGCCAGTGGA | -1400 |
| ***L3MBTLp3*** | TGTGACAGTCTCTCCAGCTC | CTGATGGTGGTGGTGTTG | -900 |
| ***L3MBTLp1*** | CATTTAGACTCCACAATGACCT | CAGTCCTATCCAGCTCAAGG | -350 |
| ***L3MBTL-1*** | CTTGTGTGCAGGTGGTTG | TGGCTGGCTGAGGACTT | 0 |
| ***IGF2RAqPCR-1*** | CCAGAGAGATTAAATGACTTGC | GAAGTTTCTGAAAGCCTACCTT | -40000 |
| ***IGF2RAqPCR*** | GCTGCTGTGTGTGTAGTTCCT | TGCCTCTCCTTTCCTTCTT | -38500 |
| ***IGF2RAqPCR-2*** | TATGGTATCTCCTCACAAGTGG | CACACAGTAGGTACTAGCAATGG | -37000 |
| ***IGF2RqPCRp2*** | GAGAAGCATCATGTAGAAATTG | ACTCTGCAATCCAGTGATACT | -1500 |
| ***IGF2RqPCREx1*** | GATGCTGTTGATATTGAAAATC | CGAAGTATTCCGATTAAATGC | 0 |
| ***IGF2RqPCRin1*** | CCAAATGGAAGCCAGTAACT | ATGCTACAGCTCACAATGC | +1700 |
| ***Albumin_qPCR*** | CAGATGCTATTACTGGAAAGTA | GGATTCAGCCAAGTAGAAGA | +50 |

**Supplementary Table 6:** Relative enrichment of histone modifications at *M. domestica* imprinted loci

| ***L3MBTL*** | | |
| --- | --- | --- |
| Distance from TSS | H3K9 trimethylation | H3K4 dimethylation |
| -2700 | 0 | 1.163231 |
| -2000 | 0.116399 | 2.05930867 |
| -1400 | 0 | 1.47543 |
| -900 | 0 | 0.33624467 |
| -350 | 0.30046333 | 1.72873167 |
| 0 | 0.55712867 | 2.13965433 |
|  |  |  |
| ***IGF2R*** | | |
| Distance from TSS | H3K9 trimethylation | H3K4 dimethylation |
| -40000 | 0.2 | 2.82446883 |
| -38500 | 0.5180255 | 4.40373283 |
| -37000 | 0.2 | 3.1737205 |
| -1500 | 0.2 | 0.87235417 |
| 0 | 0.2 | -0.9346188 |
| 1700 | 0.2 | 0.89426483 |
|  |  |  |
| ***HTR2A*** | | |
| Distance from TSS | H3K9 trimethylation | H3K4 dimethylation |
| -2600 | 0.06773193 | 0.305863 |
| -1000 | 0.03596055 | 2.00495467 |
| -300 | 0.00011659 | 1.848201 |
| 400 | 0.08262711 | 1.77632667 |
